# Supplementary material for: Production of Long Fermentation Bread with Jabuticaba Peel Flour Added: Technological and Functional Aspects and Impact on Glycemic and Insulinemic Responses
Source: Foods. 2024 Sep 11;13(18):2878. doi: 10.3390/foods13182878 (PMC11431777; doi:10.3390/foods13182878)
Supplement: Supplementary file 1 [file foods-13-02878-s001.zip › foods-3150438-supplementary.pdf]

Supplementary Material

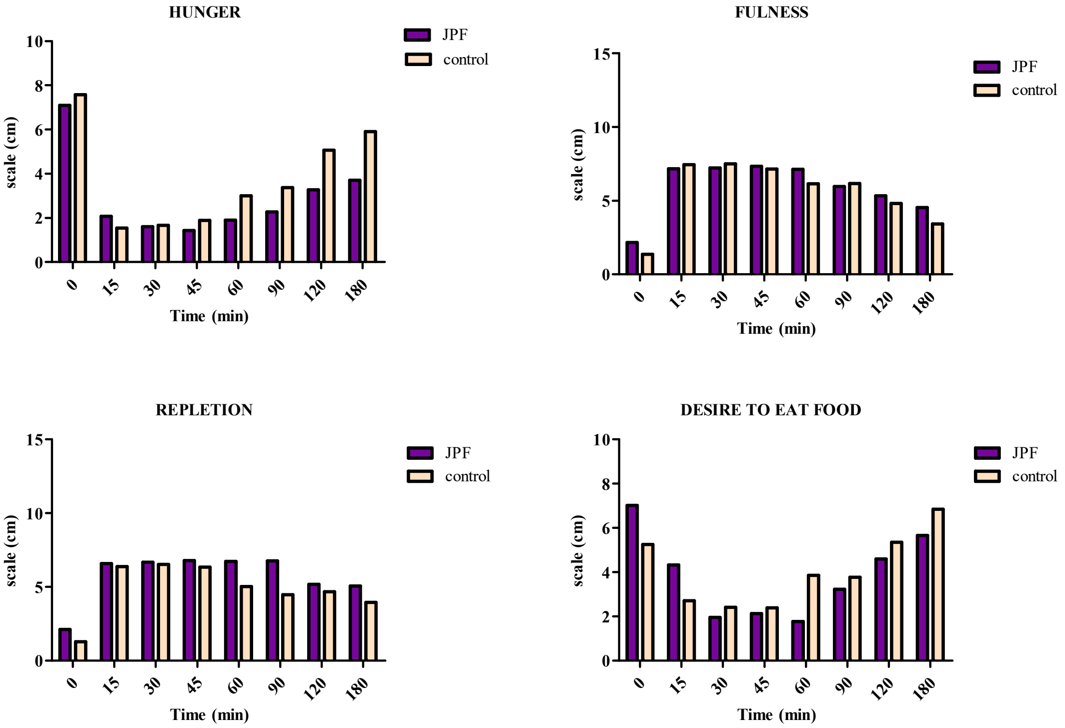

Figure S1 - Subjective appetite profile of healthy individuals after consumption of control bread or bread with jabuticaba peel flour.
